# Imperceptible magnetoelectronics

**DOI:** 10.1038/ncomms7080

**Published:** 2015-01-21

**Authors:** Michael Melzer, Martin Kaltenbrunner, Denys Makarov, Dmitriy Karnaushenko, Daniil Karnaushenko, Tsuyoshi Sekitani, Takao Someya, Oliver G. Schmidt

**Affiliations:** 1 Institute for Integrative Nanosciences, Institute for Solid State and Materials Research (IFW Dresden), Helmholtzstrasse 20, 01069 Dresden, Germany; 2 Electrical and Electronic Engineering and Information Systems, University of Tokyo, 7-3-1 Hongo, Bunkyo-ku, Tokyo 113-8656, Japan; 3 Exploratory Research for Advanced Technology (ERATO), Japan Science and Technology Agency (JST), 2-11-16 Yayoi, Bunkyo-ku, Tokyo 113-0032, Japan; 4 The Institute of Scientific and Industrial Research (ISIR), Osaka University, Mihogaoka 8-1, Ibaraki, Osaka 567-0047, Japan; 5 Material Systems for Nanoelectronics, Chemnitz University of Technology, Reichenhainer Strasse 70, 09107 Chemnitz, Germany; 6 Center for Advancing Electronics Dresden, Dresden University of Technology, Helmholtzstrasse 10, 01062 Dresden, Germany

## Abstract

Future electronic skin aims to mimic nature’s original both in functionality and appearance. Although some of the multifaceted properties of human skin may remain exclusive to the biological system, electronics opens a unique path that leads beyond imitation and could equip us with unfamiliar senses. Here we demonstrate giant magnetoresistive sensor foils with high sensitivity, unmatched flexibility and mechanical endurance. They are <2 μm thick, extremely flexible (bending radii <3 μm), lightweight (≈3 g m^−2^) and wearable as imperceptible magneto-sensitive skin that enables proximity detection, navigation and touchless control. On elastomeric supports, they can be stretched uniaxially or biaxially, reaching strains of >270% and endure over 1,000 cycles without fatigue. These ultrathin magnetic field sensors readily conform to ubiquitous objects including human skin and offer a new sense for soft robotics, safety and healthcare monitoring, consumer electronics and electronic skin devices.

## Abstract (web summary)

Birds and many other animals can sense the Earth’s magnetic field, but not human beings. Here, Melzer *et al*. develop a type of artificial skin based on giant magnetoresistive sensor foils with micrometre thickness, which can be stretched up to >250% without sacrifices in device performance.

## Graphical abstract

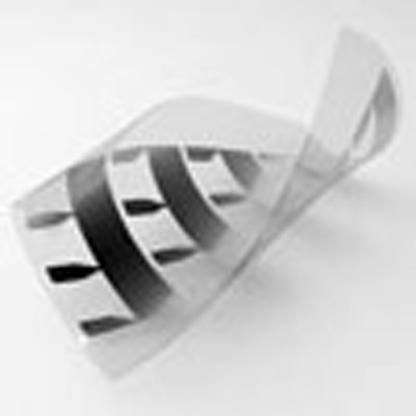

Electronics of tomorrow will be imperceptible and will form a seamless link between soft, living beings and the digital world^1^. Inspired by natures antetype, electronic skin is an intriguing technological platform already able to perceive temperature changes^2^,^3^, mimic the sensation of touch^4^,^5^,^6^, monitor and display physiological conditions^7^,^8^, communicate wirelessly, and harvest and store energy for autonomous operation^9^,^10^,^11^,^12^,^13^. Concepts that enable self-healing^14^ will lead to durable, multifunctional artificial skin. Other functionalities, however, especially those that are unfamiliar to human beings, are not addressed so far.

Magnetoception is a sense, which allows bacteria, insects and even vertebrates such as birds and sharks, to detect magnetic fields for orientation and navigation^15^,^16^. Humans are however unable to perceive magnetic fields naturally, but electronic skin could soon help to bridge this gap. Soft, flexible and transient smart sensorics^17^ to monitor physiological conditions are at the forefront of multidisciplinary research efforts bridging materials science, electrical engineering and medicine. Magnetosensorics is a versatile tool to assess mechanical movements also *in vivo*; foreseeable applications include real-time monitoring of artificial muscles, joints or valves of the heart to diagnose early stages of dysfunctions. Such advanced applications require very specific mechanical properties of the sensing elements, such as bending radii <10 μm, stretchabilities exceeding 100%, as well as a sensitivity for magnetic fields below 100 Oe, all of which are met by our ultrathin magnetic sensors, making them ideally suited for wearable, yet unobtrusive and imperceptible orientation and manipulation aids.

Here we go beyond imitating the features of human physiology and introduce e-skins with a magneto-sensory system, which equips the recipient with a ‘sixth sense’ able to perceive the presence of static or dynamic magnetic fields. We demonstrate an on-skin proximity detection systems for touchless human–machine interaction, motion and displacement sensorics applicable for soft robots^18^,^19^ or functional medical implants^20^,^21^, as well as magnetic functionalities for epidermal electronics^7^. In this work, we construct highly sensitive giant magnetoresistive (GMR) sensor elements on ultrathin, 1.4 μm polyethylene terephthalate (PET) foils. Weight and flexibility are key figures of merit for large area electronics or robotic skin^7^,^22^, as they critically influence the mechanical response and perception of the artificial sensory system. With just about 1.5 μm total thickness, imperceptible magneto-electronic foils are light (≈3 g m^−2^) and unmatched in flexibility; they are operable with radii of curvature below 3 μm, yet are highly durable and can withstand severe crumpling without any performance degradation. These are prerequisites for intimate contact with soft, biological tissue or organs and complex, arbitrarily shaped three-dimensional free forms. Despite their imperceptible design, our GMR sensors exhibit high sensitivities of up to 0.25% per Oe, identical to their counterparts on rigid Si/SiO_2_ wafer substrates. Biological skin is soft and flexible but also stretchable, a feature that is most desirable for the artificial equivalent. Imperceptible electronic foils^11^,^22^,^23^ offer an elegant route to facilitate very high levels of strain without any sacrifices in device performance by a facile postfabrication transfer step onto a prestrained elastomer. We demonstrate magneto electronics that can reversibly attain tensile strains up to 270%, nearly a tenfold increase over previously reported concepts^24^. We prepare magneto-sensitive elements that are compliant to uniaxial and biaxial deformation, creating a universal potential for applications in stretchable electronic systems. Moreover, our devices are remarkably stable, withstanding 1,000 stretch cycles without fatigue.

## Results

### Imperceptible magnetoresistive sensor skin

**Figure 1:**
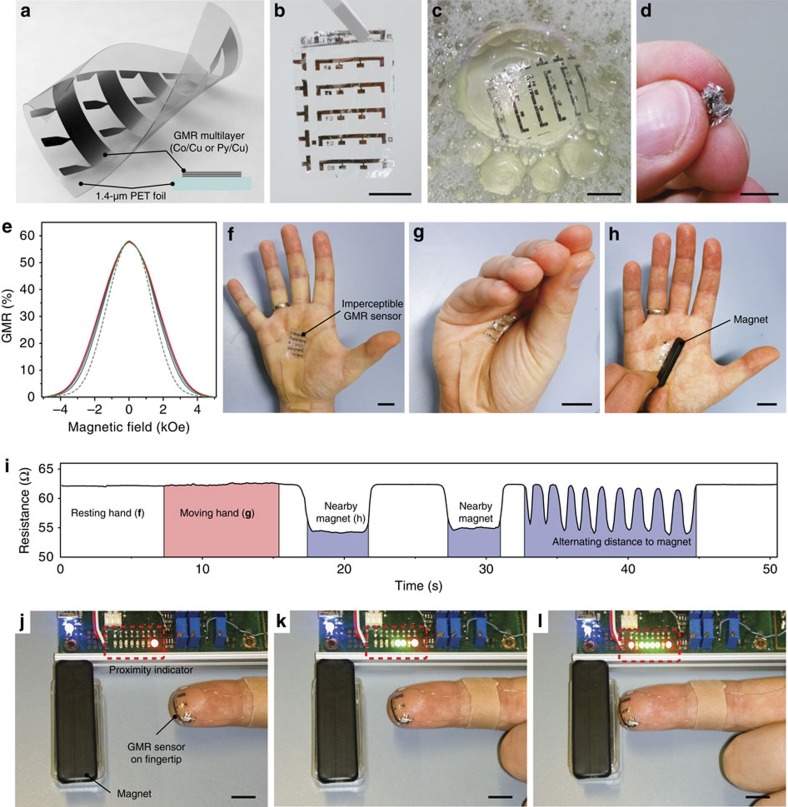
GMR multilayers on ultrathin PET. (**a**) Diagram of an imperceptible magnetic sensor foil. Cross-section of the GMR layer system (inset). (**b**) Free-standing array of five Co/Cu multilayer elements on 1.4-μm-thick PET foil. (**c**) Ultralight (3 g m^−2^) sensor array floating on a soap bubble and (**d**) crumpled between fingertips. Scale bars, 10 mm. (**e**) GMR characteristics of one Co/Cu first maximum element as prepared (green curve, *R*=16.9 Ω), after attaching to the palm and folding (blue curve, *R*=17.0 Ω), and after crumpling as shown in **d** (red curve, *R*=17.9 Ω). Comparison with a reference sample on rigid silicon (dashed grey curve, *R*=16.2 Ω). Corresponding measurements on the other elements in the array gave similar results. (**f**,**g**,**h**) Imperceptible GMR sensor array on a human palm with one element connected to a readout circuit during rest, moving the hand and in proximity to a permanent magnet. Scale bars, 20 mm. (**i**) Recorded resistance of the sensor element for **f** through **h** and Supplementary Movie 3. (**j**,**k**,**l**) On-skin magnetic proximity sensor using a Co/Cu second maximum GMR element on the fingertip connected to a linear array of light-emitting diodes (LEDs) (dashed red frame) as touchless interface. Scale bars, 10 mm (see Supplementary Movie 4).

GMR multilayer elements (Co/Cu and Py/Cu multilayers; Py=Ni_81_Fe_19_) are directly fabricated on 1.4 μm ultrathin PET foils (Supplementary Figs 1 and 2). The structure of an array of five sensor elements is schematically shown in Fig. 1a, details on the fabrication process are found in the Methods section. The substrate foil is commodity scale, commercially available PET (Mylar 1.4 CW02) that is fully compatible with lithography and lift-off processes, which allows for accurately patterned individual devices, yet large area low-cost manufacture. The ultrathin substrate is temporarily self-adhered to a reusable support (see Methods for details) for ease of handling. After device fabrication, the imperceptible sensor skin is readily peeled off the handling support without causing damage, as demonstrated in Fig. 1b with a free-standing ultrathin (1.5 μm total thickness) array of five lithographically structured Co/Cu GMR multilayer elements. The extreme light weight and compliant nature of the sensor elements is demonstrated in Fig. 1c, where an array of magnetic field sensors is floating on a fragile, spherical soap bubble. Our sensor foil is highly flexible; it can be completely scrunched up and rubbed between fingertips multiple times (Fig. 1d and Supplementary Movie 1) without signs of performance degradation. This is corroborated by GMR characteristics (Fig. 1e) recorded for the as-fabricated sensor and after crumpling as in Fig. 1d, showing that even severe mechanical stress has virtually no influence on the device characteristics. In spite of the 1.4-μm PET foils’ rough surface (29 nm root mean square as determined by atomic force microscopy, Supplementary Fig. 3), our ultrathin magnetic sensor foils perform equal to reference samples fabricated on smooth, rigid Si/SiO_2_ waver substrates. Instead, we take advantage of the enhanced adhesion of thin metal films on rough polymer surfaces^22^, which leads to an improved mechanical resilience. The prepared elements reveal a high GMR ratio of 57.8% at room temperature, which is a typical value for Co/Cu multilayers^25^. Apart from a slightly increased saturation field, the GMR signal on PET is very similar to the reference data on Si/SiO_2_ and exhibits the same GMR magnitude. The electrical resistance of the flexible sensors (16.9 Ω) is comparable to their rigid counterparts (16.2 Ω). Motivated by these results, we continued to prepare Co/Cu and Py/Cu multilayers coupled in the second antiferromagnetic maximum on ultrathin PET foils. These GMR systems have more stringent requirements with respect to substrate quality and deposition parameters, but exhibit much higher sensitivities to small magnetic fields^26^,^27^. This is especially desirable for smart skin, biomedical and orientation applications^28^,^29^,^30^. The magneto-electric characterization of the second maximum GMR multilayers (Supplementary Fig. 4) reveals that even these highly sensitive elements prepared on ultrathin PET behave very similar to their rigid counterparts prepared on Si/SiO_2_ silicon wafers, which strengthens the potential of imperceptible magnetoelectronics for a wide field of GMR and spintronic systems.

Imperceptible magnetoelectronics is readily worn directly on the palm of a volunteer’s hand as demonstrated in Supplementary Movie 2. Here, a set of Co/Cu first maximum GMR sensors intimately conforms to the inner hand and unobtrusively follows the motions and deformations of the skin when the hand is moved. A sensor element is electrically contacted with thin copper wires (Fig. 1f) by using conductive silver paste and the resistance of the on-skin sensor is recorded while moving the fingers, opening and closing the hand (Fig. 1g), applying a magnetic field with a permanent magnet and alternating the distance to the magnet (Fig. 1h). The resistance plotted in Fig. 1i and Supplementary Movie 3 shows a small, noise-induced fluctuation during the motion of the hand (which amounts to <0.3%), whereas the field of the approaching permanent magnet induces a strong resistance drop of about 13% at it’s nearest position. Altering the distance between the permanent magnet and the on-skin sensors results in a corresponding change in magnetoresistance. Note that magnetic saturation of the Co/Cu multilayer element is not reached, as the full bandwidth of the sensor is not used here. Electronic skin on fingertips^31^,^32^ is especially attractive for precise input and as communications interface due to our finger’s fine motor skills. In Fig. 1j,k,l and Supplementary Movie 4, we demonstrate an on-skin magnetic proximity sensor where a single GMR element is attached to a fingertip and connected to a readout circuit (see Supplementary Fig. 5 for details). The presence of any kind of magnetic field can thus be detected by simply pointing the finger towards it and its strength is visualized by an array of light-emitting diodes. Unlike optical sensors, no line-of-sight between the sensor and the magnetic field emitter is required. This allows transmitting ‘magnetic messages’ through all non-magnetic objects such as safety enclosures, displays or even walls. The encoding can be realized both statically via permanent magnets as well as dynamically simply with current-driven wirings. In all those demonstrations, the imperceptible GMR sensors require no encapsulation or capping layers.

### Stretchable magnetic field sensors

**Figure 2:**
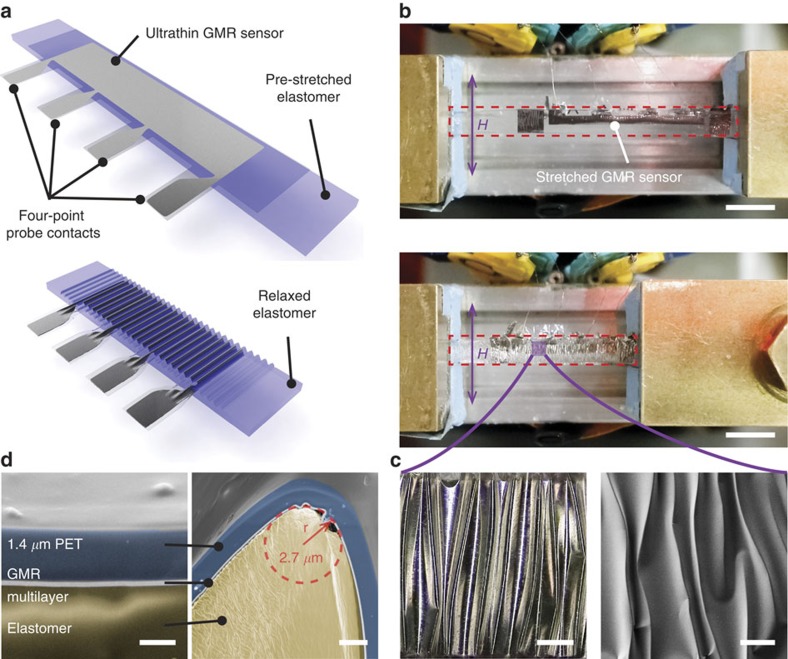
Stretchable GMR sensors. (**a**) Illustration of stretchable magnetoelectronics. A multilayer GMR element on ultrathin PET is laminated face down onto a prestretched stripe of sticky rubber tape. Four contact pads are reaching beyond the tape (top). Relaxing the elastomer results in out-of-plane wrinkling of the sensor foil and enables re-stretching (bottom). (**b**) Py/Cu second maximum sample mounted to the *in situ* stretching stage fully elongated (top) and compressed by 50% (bottom). The pink arrow indicates the axis of the applied magnetic field. Scale bars, 5 mm. (**c**) Optical microscopy (scale bar, 200 μm) and SEM. (scale bar, 100 μm) top-view images reveal the wrinkle structure of the sensor surface in its compressed state. (**d**) Cross-sectional SEM images of the sensor foil laminated to the rubber tape. The GMR nanomembrane is encapsulated between the ultrathin PET and the stretchable adhesive tape. Some parts of the magnetoresistive foil on the tip of the buckles are bent into radii of curvature of <3 μm (right). Scale bars, 1 μm (left), 2 μm (right).

**Figure 3:**
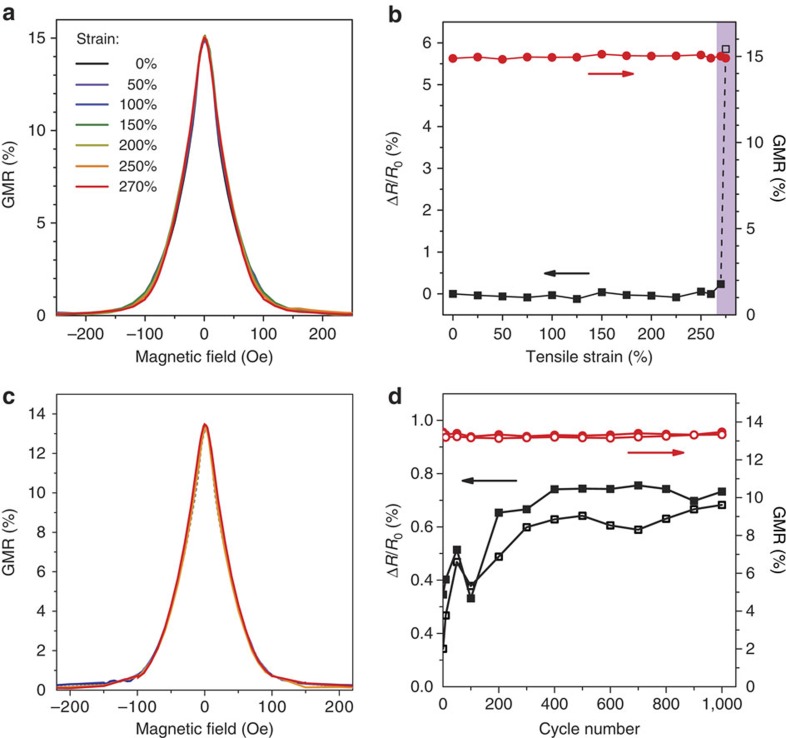
Results of stretching experiments. (**a**) GMR curves recorded for strains from 0% to 250% in increments of 50%, plus 270%. (**b**) GMR magnitude (red dots) and resistance change normalized to 0% strain (black squares, *R*_0_=9.7 Ω) as a function of applied strain. The shaded region indicates overstretching with plastic deformation of the sensor foil. Reliability on cyclic loading. (**c**) GMR curves of a Py/Cu second maximum element at 50% strain (blue) and 100% strain (red); first cycle in light shades, cycle 1,000 in strong shades. The characteristic of the as-prepared sample is plotted in dashed grey. (**d**) GMR magnitude (red dots) and resistance change normalized to the as-prepared sample (black squares, *R*_0_=10.0 Ω) at 50% strain (open symbols) and 100% strain (closed symbols) as a function of cycle number.

Artificial skin components should not only be flexible, but ideally stretchable in a mode that enables high multidimensional deformations. Our ultrathin magnetoelectronic elements can be made stretchable in a one-step postfabrication transfer process by laminating them onto a prestretched elastomer (3 M very high bond (VHB)), as illustrated in Fig. 2a for uniaxial strain. When the elastomer is allowed to relax, out-of-plane wrinkles form in the sensor foil, which enables stretching of the device in the direction of the initial deformation of the elastomer^33^. The magnetosensitive capabilities of the presented elements are not affected by this process, as shown in Supplementary Fig. 6. A top view of a stretchable GMR-sensing element, mounted into the stretching stage for *in situ* measurements, is provided in Fig. 2b. Highly sensitive Py/Cu second maximum multilayer elements are operated between the pole shoes of an electromagnet and contacted for a four-point resistance measurement (see Methods section for details on contacting). The axis of the applied magnetic field is perpendicular to the sensor stripe (along the wrinkles). Optical and scanning electron microscopy (SEM) top-view images in Fig. 2c reveal the wrinkle topology of the sensor, laminated to the rubber tape, while undergoing 50% compression. The GMR elements are attached face down on the pre-stretched elastomer, which in turn acts as an encapsulation for the functional magnetic layer between the stretchable tape and the ultrathin PET foil, as visualized by SEM imaging of the sample’s cross-section (Fig. 2d) prepared by focused ion beam (FIB) milling. The maximum compression and therefore stretchability of the sensors is determined by the pre-strain of the elastomer before lamination and limited by the packing density of the out-of-plane folds^22^. We analysed cross-sectional SEM images of samples prepared with a high uniaxial pre-strain (Fig. 2d right), where some parts of the magnetoresistive foil on the tip of the buckles are bent into radii of curvature of <3 μm, while the sensor not only remains functional, but also maintains its full performance (a top-view SEM image, indicating the locations of the FIB cuts is provided in Supplementary Fig. 7). GMR curves recorded at different tensile strain levels up to 270% along the direction of pre-strain are congruent with each other, as presented in Fig. 3a (Microscopy images of wrinkles at different strains can be found in Supplementary Fig. 8). At this point, the PET foil with the GMR layer is fully elongated and the wrinkles vanish. Supplementary Movie 5 shows the stretching of a sample until the wrinkles disappear; confocal microscopy images of a sample at the maximum strain and just below are provided in Supplementary Fig. 9. The progression of the GMR magnitude and the relative resistance change of the sensor are plotted as a function of the uniaxial deformation in Fig. 3b. Both values remain unchanged up to 270% strain (relative s.d.: RSD_R_<0.1%; RSD_GMR_<0.6%). Once the foil is stretched beyond it’s fully flat state, the resistance increases due to some plastic deformation of the flattened GMR layer. However, by overstretching the GMR element by several per cent, here from 270% to 275%, the magnitude of the GMR effect is not reduced (shaded region in Fig. 3b) and the device remains fully operational. Results from a similar stretching experiment on a sample fabricated with a lower pre-strain are given in Supplementary Fig. 10, including also a GMR curve on overstretching. As both the GMR magnitude and the sensor’s resistance are invariant to stretching, no additional strain calibration is necessary. Our approach thus eliminates a major drawback of previous approaches to stretchable GMR multilayers^26^,^34^.

### Long-term performance on cyclic stretching

We performed cyclic loading experiments to demonstrate the reliability of our imperceptible, highly stretchable magnetoresistive elements. Wrinkled Py/Cu second maximum multilayer sensors were prepared using a pre-strain of 150%. The strain region for the repeated loading and unloading was set from 50% to 100%, to meet the maximum permanent operation limits of the test setup and to avoid slacking due to the viscoelasticity of the elastomeric tape during this long-term test. This strain region typically meets the demands for most on-skin and *in vivo* operations. GMR characteristics taken at the low- and high-strain reversal points for the first and 1,000th loading cycle are displayed in Fig. 3c and show no signs of fatigue. The recorded traces are congruent with each other and to the control measurement of the as-prepared sample (at 0% strain). Figure 3d plots the GMR magnitude and relative resistance change (normalized to the as-prepared sample) for the high (100%) and low (50%) strain reversal points versus cycle number. The GMR values remain at their high level throughout the 1,000 loading cycles, and even the electrical resistance of the nanomembrane stays nearly unchanged (far less than 1% change over 1,000 cycles). Although a slight resistance increase is observed over the first 200 cycles, saturation sets in and no further fatigue occurs. This is remarkable for a fully functional sensor element, as such a long-term stability is rarely observed even for stretchable conductors^35^. Our results prove that imperceptible GMR sensors are rugged and very durable, a prerequisite for ‘real world’ electronic skin applications.

### Biaxial stretchability on a soft diaphragm

**Figure 4:**
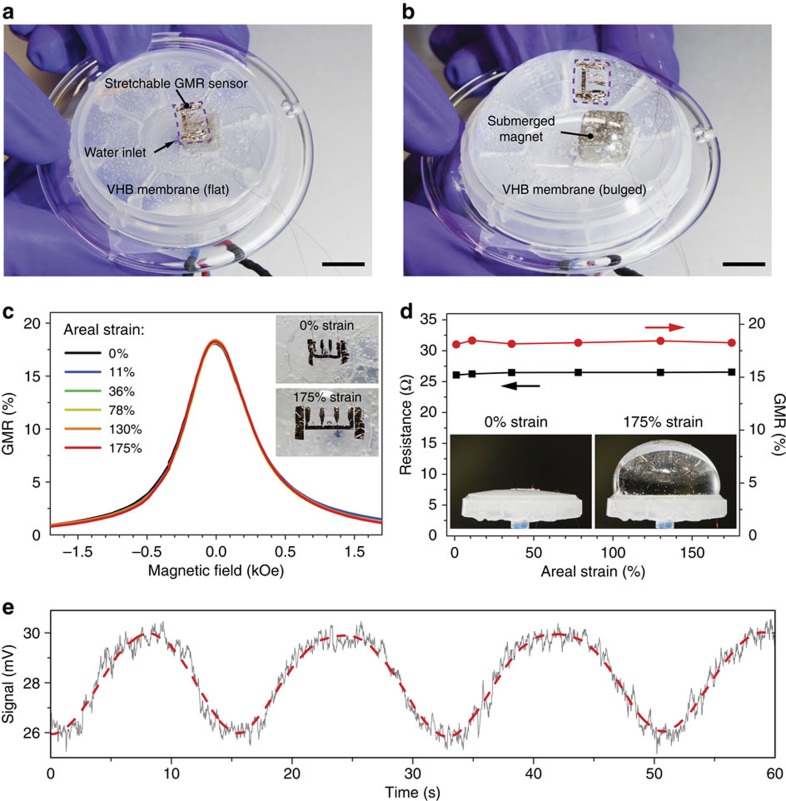
Biaxial stretching on a soft diaphragm. (**a**,**b**) Biaxially stretchable Co/Cu second maximum GMR sensor on a VHB membrane spanned over a plastic tray to create a sealed chamber. Water is pumped through the inlet to inflate the VHB membrane and stretch the sensor along both lateral directions. A permanent magnet was fixed inside the water chamber, to enable the dynamic detection of diaphragm inflation/deflation cycles. The flat and fully inflated states are shown in the left and right inset, respectively. (**c**) GMR curves recorded for different areal strains, as stated in the legend. The left and right insets show the biaxially wrinkled sensor at 0% and 175% strain. (**d**) GMR magnitude (red dots) and sensor resistance (black squares) as a function of applied areal strain. The strain was estimated from side-view photographs of each inflated state (Supplementary Fig. 11). (**e**) Sensor signal for a pulsating diaphragm for the dynamic magnetic detection of its inflation/deflation (see Supplementary Movie 6). The dashed line is a smoothed graph to guide the eye.

Using biaxial pre-strained membranes, our GMR sensors can be stretched in all lateral directions simultaneously. We demonstrate this by laminating an imperceptible sensor on an expanding VHB tape diaphragm (Fig. 4a,b) that is covering the circular opening of a plastic tray and forms a waterproof chamber. The sensor is attached while the VHB membrane is strongly inflated with water pumped into the cavity (Supplementary Movie 6), causing the tape to be highly prestrained biaxially. Figure 4c,d show the GMR signal and resistance evolution of the Co/Cu second maximum sensor on the diaphragm at different inflation states, spanning from planar (0%) to 175% areal strain. As for the uniaxial case, the GMR response as well as the sensor’s resistance remain stable and are not adversely affected by imposing significant levels of biaxial strain. A picture sequence showing the inflation and the senor for all measured strain levels is given in Supplementary Fig. 11.

The geometry of an expanding diaphragm is of particular interest, as it represents several potential applications for biaxially compliant magnetic sensors, for example, soft diaphragm actuators made of electro-active polymers^36^,^37^, or multifunctional medical implants on muscular biological tissue^21^. We can readily monitor the inflation state of the diaphragm with our biaxially stretchable sensor by placing a permanent magnet in the water chamber (Fig. 4b, Supplementary Fig. 12 and Supplementary Movie 6). The stretchable sensor is perfectly monitoring the pulsation of the diaphragm (Fig. 4e) as the distance between the magnet and the compliant magnetoresistive foil changes during inflation and deflation.

## Discussion

For stretchable electronics in general, the interface from the soft elements to the rigid parts, such as power sources or signal processing units, is a very demanding aspect for application and characterization. Especially in the case of high tensile deformations or repeated loadings, the electrical contact points of wires to the contact pads of the stretchable elements tend to fail much earlier than the occurrence of fatigue of the actual device under investigation^24^. With the method used in this work (contact pads on ultrathin PET foil that reach sideways beyond the stretchable support), a reliable four-point characterization of the functional elements under highest applied strains and repeated loading cycles was achieved. The soft, yet not expanding, pads could easily be contacted with copper wires using conventional conductive silver paste without any heat treatment.

Furthermore, we attribute the outstanding resilience of the presented imperceptible and stretchable GMR element against high mechanical deformations in particular, to the ductile properties of copper in our Co/Cu and Py/Cu GMR stacks. As a first approximation for the peak strain in the wrinkled metal layer on the ultrathin PET film, an established model for the bilayer bending strain^38^ can be applied. This computes the strain *ε*_top_ in a rigid film (elastic module: *Y*_f_, thickness *d*_f_) on a softer substrate (elastic module: *Y*_s_, thickness *d*_s_) on bending to a radius *R* via


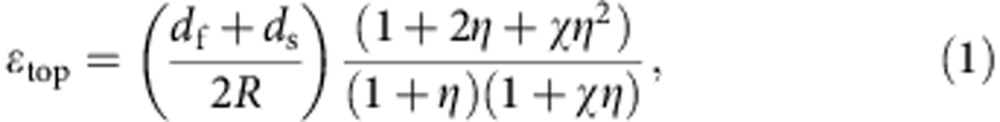


with *η*=*d*_f_/*d*_s_ and *χ*=*Y*_f_/*Y*_s_. The properties of the PET foil are *Y*_s_=3 GPa and *d*_s_=1,400 nm. We estimate the elastic module of the GMR layer (Co/Cu in the first maximum: *d*_f_=111 nm) by its material composition (60-nm Cu: *Y*_Cu_=131 GPa; 51-nm Co: *Y*_Co_=211 GPa) to be 171 GPa. For the measured radius of curvature (<3 μm), induced by wrinkling in the relaxed state (Fig. 2d), the calculation according to the equation above results in the strain of ~5.6%. However, this value is above the fracture strain of the GMR layer. Different effects are considered to reduce the actual strain, for example, an increased strength observed in multilayer thin films^39^ or the shifting of the neutral mechanical plane towards the active element by the sandwiching VHB elastomer. Furthermore, for the case of compressive strain on the GMR layer underneath the buckles, the FIB cuts revealed additional microscopic wrinkling of the magnetic nanomembrane on the PET (Fig. 2d, right side). Beyond that, we observe a much lower curvature in the wrinkle valleys (Fig. 2c, right side), which would also reduce tensile strains. A maximum peak strain of 0.5% in the GMR layer would allow a bending radius of below 40 μm according to equation (1).

In conclusion, we develop imperceptible and highly sensitive magnetic field sensors with unique mechanical properties and demonstrated the remarkable potential of this technology for smart skins, consumer goods and medical implants. These ready-to-use sensing elements extend the cognition of electronic skin systems to a medium, which, by no means, can be detected by human beings. They are ultra-lightweight, conform to arbitrary surfaces and seamlessly follow deformations or distortions without performance degradation. For the emerging field of stretchable magnetoelectronics, the GMR sensors presented here outperform all previously introduced elements in terms of stretchability, reliability and fabrication potential by a multiple. These versatile features are imparted to the magnetoelectronic devices by their ultrathin and flexible, yet robust polymeric support. The sensor layout used in the present work was chosen for ease of precise characterization. As photolithographic patterning of the magnetoelectronic nanomembranes on the ultrathin plastic foils was successfully applied in the present work, the device structure can be adapted and scaled to meet the requirements for specific applications and design concepts. Patterning defined meander structures, for instance, can adapt the electrical parameters of individual sensing elements to the requirements of a specific signal processing electronics. Preparing the GMR elements into a bridge configuration would allow for a differential signal and may compensate for temperature effects. The utilization of spin valve layer stacks instead of GMR multilayers can result in even higher sensitivities to small magnetic fields. Future work will focus on optimizations to interface electrically and mechanically with other electronic components enabling, for example, wireless readout and remote sensing. The integration of magnetoelectronics with ultrathin functional elements such as solar cells^11^, light-emitting diodes^23^, transistors^40^, as well as temperature and tactile sensor arrays^22^, will enable autonomous and versatile smart systems with a multitude of sensing and actuation features. Detailed mechanical and electrical modelling will further guide sensor improvement. We foresee our work to inspire a diverse number of devices that will benefit from a ‘sixth sense’ magnetoception.

## Methods

### GMR multilayers on ultrathin PET

PET foil (1.4 μm thin; Mylar 1.4 CW02, Pütz GMBH+Co. Folien KG) where temporally self-adhered to a reusable, 125-μm-thick polymer support coated with a thin layer of poly(dimethylsiloxane). Van der Waals adhesion allows both for device handling during lab-scale fabrication processes and subsequent detachment and transfer of the final element to other substrates and surfaces. A positive ultraviolet lithography lift-off process was performed using AZ 5214E resist (AZ Electronic Materials) and an MA56 mask aligner (SÜSS Micro Tech), to define individual sensor stripes of 1 × 16 mm^2^ with a four-probe contact geometry. Giant magnetoresistive multilayers comprising of Co(1 nm)/[Co(1 nm)/Cu(1.2 nm)]_50_ (Co/Cu first maximum), Co(1 nm)/[Co(1 nm)/Cu(2.2 nm)]_50_ (Co/Cu second maximum) or Py(1.5 nm)/[Py(1.5 nm)/Cu(2.3 nm)]_30_ (Py/Cu second maximum) were grown by magnetron sputter deposition at room temperature (Py denotes soft magnetic permalloy: Ni_81_Fe_19_). The sputter parameters were as follows: base pressure, 7.0 × 10^−7^ mbar, Ar sputter pressure, 7.5 × 10^−4^ mbar and deposition rate, 2 Å s^−1^. After the lift-off process, the ultrathin sensor foil could be readily peeled from the handling support without causing damage and attached on the skin or any arbitrarily shaped surface. Electrical contacts were realized using thin (100 μm) enameled copper wires and G 3303B conductive silver paste (Plano).

### Stretchable GMR elements

VHB 4905 F (3 M) adhesive tape was used as the elastomeric support for the preparation of stretchable GMR sensors. Stripes of 5 × 50 mm^2^ were pre-stretched by 600% longitudinal (if not stated differently). Individual elements with elongated contacts were cut from a prepared array of Py/Cu second maximum multilayer sensors and attached with the metallized side onto the sticky surface of the pre-stretched VHB so that the four contact pads reach sideways beyond the tape. After removing the polymeric handling support, the multilayer element was contacted with four copper wires and conductive silver paste. As the pre-strain is released, wrinkles occur on the ultrathin PET foil with the buried magnetic nanomembrane, which are aligned along the short axis of the VHB stripe. The viscoelastic behaviour of the VHB tape prevents a relaxation to the initial length from high prestrains. Consequently, a 600% prestretched tape allows the sensor to be stretched by 270%. In the stretching experiments, the length of the sensor element after a relaxation period of 1 day is defined as the reference length and corresponds to 0% strain.

### GMR sensors on expanding diaphragm

A hole was drilled into the bottom of a 55-mm-diameter circular plastic tray to fix a fluidic tubing and a permanent magnet was glued into the tray at a central position. A piece (50 × 50 mm) of VHB 4905 F (3 M) was spanned over the circular edge of the tray with a strain of about 50% × 50% and adhered around the outer edge to seal the inner camber housing the permanent magnet. The back end of the fluidic tubing was connected to a 50-ml syringe to pump water into the sealed chamber and inflate the VHB membrane. At a highly inflated state, a Co/Cu second maximum GMR sensor on ultrathin PET foil, contacted on the two outmost contact pads, was attached face down to the top of the thus created water dome. On deflating the diaphragm using the syringe, the sensor was biaxially compressed and wrinkled accordingly. In this setup, the sensor resistance gives a measure for the distance to the permanent magnet, and hence the inflation of the diaphragm controlled by the water syringe. The GMR characterizations of the sensor on the diaphragm were performed with a similar setup without the permanent magnet.

### *In situ* GMR stretching setup

We tested the uniaxial stretchability of the prepared GMR-sensing elements using an automated computer-controlled *in situ* stretching stage with four-point electrical contact clips, which can be operated between the pole shoes of an electromagnet for characterization. An outline of the setup is depicted in Supplementary Fig. 13. The sample was mounted between two stretching clamps and their separation was adapted until the slack was removed. After applying the attached copper wires to the contact clips, the electrical resistance was measured while sweeping the field perpendicular to the sensor stripe with the electromagnet. For the stretchability tests, the strain was increased in steps of 10% with a rate of 100 μm s^−1^ (0.7% s^−1^) before measuring the next GMR curve. For the cyclic loading experiment, the sensor was first stretched to 1.5 times its length and then the strain was repeatedly increased and decreased by 50% (with respect to its original length) with a rate of 150 μm s^−1^ (1.25% s^−1^) and 1-s delay at the reversal points. At each tenth cycle, a new GMR curve was recorded at the two reversal points, respectively, before the loading and unloading proceeded. For the GMR characterization on biaxial stretching, the expanding diaphragm setup was placed between the magnet pole shoes instead of the stretching stage. GMR curves were recorded using a two-point resistance measurement at different inflation states, which are defined by an additional volume of 10 ml water pumped into the chamber before the next measurement.

## Author contributions

M.M. designed and fabricated the GMR sensors and conducted the experiments. M.M., M.K. and D.M. analysed the data and prepared figures with contributions from all authors. M.K., T.Sekitani. and T. Someya investigated the mechanical properties and compliancy of thin functional films on PET foils. Dmitriy K. and Daniil K. designed and assembled electronics for the demonstrators, prepared conceptual pictures and supported M.M. with experiments. M.M. and M.K. wrote the manuscript with comments from all authors. D.M., T. Sekitani, T. Someya and O.G.S. supervised the project and advised on device optimization.

## Additional information

**How to cite this article:** Melzer, M. *et al*. Imperceptible magnetoelectronics. *Nat. Commun.* 6:6080 doi: 10.1038/ncomms7080 (2015).

## Supplementary Material

Supplementary Figures Supplementary Figures 1-13

Supplementary Movie 1 Rubbing and crumpling of GMR sensors on ultra-thin PET foil. The video shows how the PET foil equipped with GMR sensors can be tightly crumpled. A comparison of the GMR characteristics before and after this recording shows that the sensors survive this harsh treatment without suffering in performance (compare green and red curve in Fig. 1e). Only the resistance is slightly increased. Please note that there is no seed or capping layer of the GMR film or any kind of encapsulation present.

Supplementary Movie 2 Array of imperceptible GMR sensors situated on the human palm. The video shows an array of five GMR multilayer elements (Co/Cu 1st maximum) on a sheet of ultra-thin PET foil, which is mounted to the palm of a human hand. The on-skin magnetic sensors can conformably follow the complex deformations imposed by the natural motion of the hand. A comparison of the performance of the most central sensor element before and after this treatment is given in Fig. 1e (compare green and blue curve), which reveals no changes.

Supplementary Movie 3 Contacted magnetic field sensor on the palm and recorded resistance. This video demonstrates the compliancy of an on-skin sensor array (Co/Cu 1st maximum) attached to a human palm with one element contacted, together with its live recorded resistance. It shows that the resistance change during the motion of the hand is low compared to the change imposed by the presence of a permanent magnet. The recorded resistance curve is also shown in Fig. 1i.

Supplementary Movie 4 Single GMR element on a fingertip as pointing magnetic proximity sensor. This video shows a single GMR element (Co/Cu 2nd maximum) attached to a fingertip and connected to an electronic circuit, to be used as an on-skin magnetic proximity sensor. A description of the self-made electronic board can be found in supplementary figure S5. The magnetic field of a permanent magnet is detected by the on-skin sensor by simply pointing the finger towards it. An array of LEDs on the electronic board indicates the strength of the field in dependence of the distance to the magnet. The experiment corresponds to the demonstration shown in Fig. 1j,k,l.

Supplementary Movie 5 Stretching of a prepared GMR sensor in the in situ characterization setup (4x time lapse). This video shows a highly sensitive stretchable GMR multilayer sample (Py/Cu 2nd maximum) mounted to the in situ stretching stage used for magneto-electrical characterizations under applied strains. It demonstrates the stretching of the sensor up to the final point, when the wrinkles disappear and the GMR layer becomes flat. Screenshots of this video are also provided in Fig. 2b.

Supplementary Movie 6 Dynamic detection of a soft diaphragm inflation using a biaxially stretchable GMR sensor. The video shows the mounting of an imperceptible sensor (Co/Cu 2nd maximum) onto the VHB membrane which is highly inflated to a diaphragm using water pressure. In the second scene, the diaphragm is pulsating (twofold time lapse) and the mounted sensor detects the inflation of the diaphragm by means of a permanent magnet fixed inside the water chamber. The respective sensor signal is displayed in real-time on an oscilloscope screen in the background. The last part shows a close-up of the sensor while it follows the deformation of the breathing diaphragm.
