# Supplementary material for: Imperceptible magnetoelectronics
Source: Nat Commun. 2015 Jan 21;6:6080. doi: 10.1038/ncomms7080 (PMC4354162; doi:10.1038/ncomms7080)
Supplement: Supplementary Figures — 1-13 [file ncomms7080-s1.pdf]

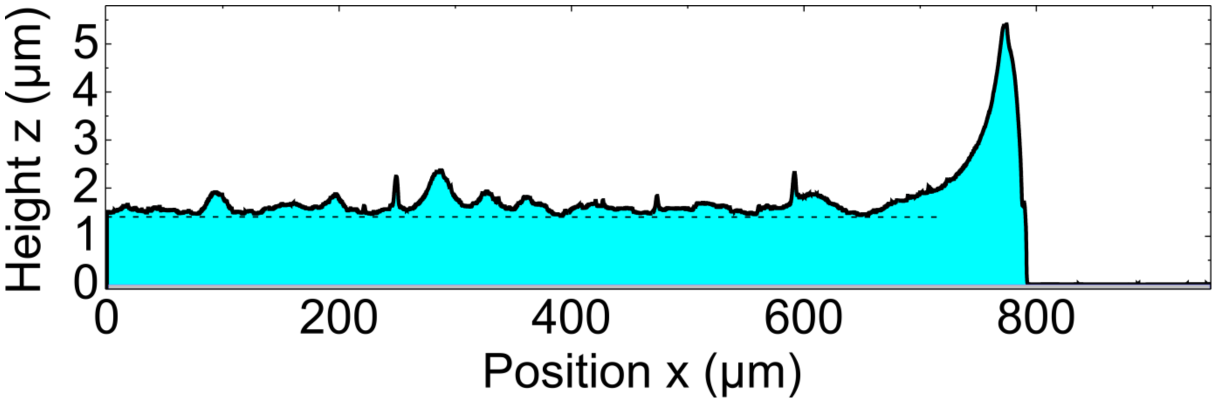

Supplementary Figure 1 | Edge profile of the PET foil. The ultra-thin PET foil was transferred to a grass slide and scanned with a DekTak 3XT surface profilometer (Bruker). The graph shows the surface profile of a 1mm scan over the edge of the foil. The average thickness between 0 and 650 µm was calculated to be 1.62 µm. The dashed line corresponds to the thickness of 1.4 µm, which is given by the manufacturer.

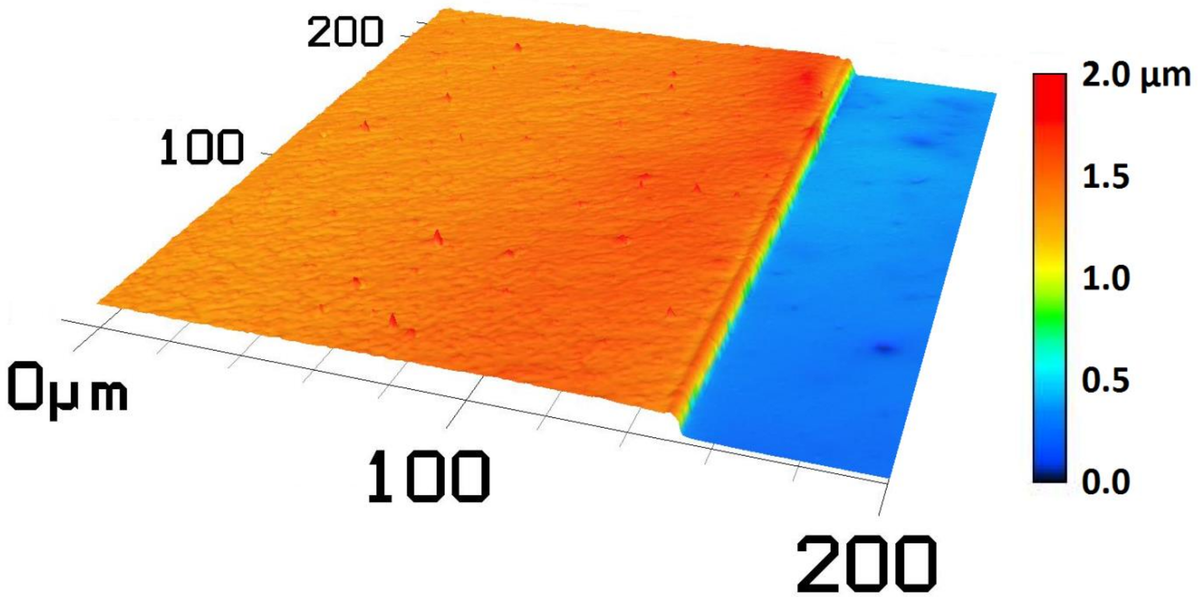

Supplementary Figure 2 | Confocal microscopy of the PET foil. The image was recorded on a VK-X200 3D confocal laser scanning microscope (Keyence) and shows the topology of an edge of the ultra-thin PET foil on top of the plastic handling substrate.

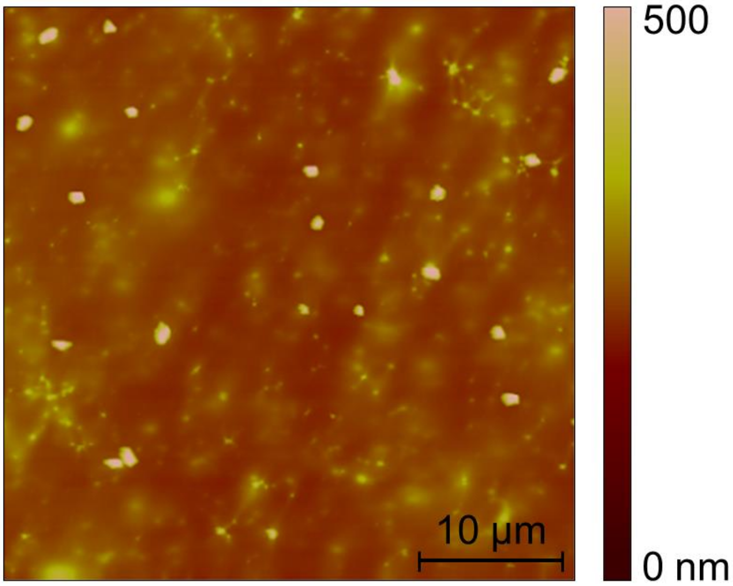

Supplementary Figure 3 | Atomic force microscopy of the PET surface. The displayed area is 40 x 40 µm². The root mean square roughness on the full image is computed to 29 nm.

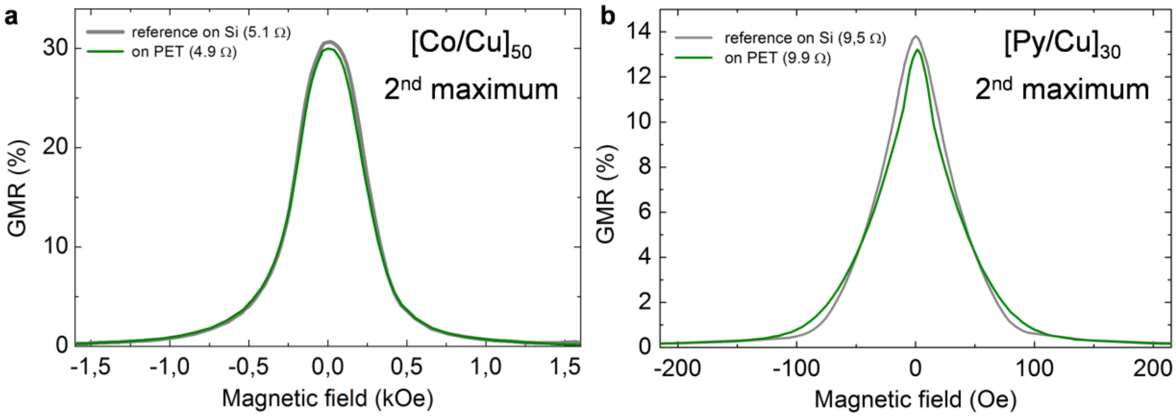

Supplementary Figure 4 | Characterization of Co/Cu and Py/Cu 2nd maximum multilayers. GMR characteristics of a Co/Cu (**a**) and Py/Cu (**b**) element, which are prepared in the second antiferromagnetic coupling maximum, on ultra-thin PET foil (green curve) and rigid silicon (gray curve) for reference. The former type of multilayer sensor was used for the on-skin magnetic proximity demonstration shown in Fig. 1j,k,l and Supplementary Video S4 due to its high GMR magnitude and a saturation field, which can easily be attained with a neodymium magnet in close distance (~1 kOe). The latter multilayer system was chosen for the preparation of stretchable sensors, as they show highest values of sensitivity to small magnetic fields, which can be applied in the *in situ* stretching setup.

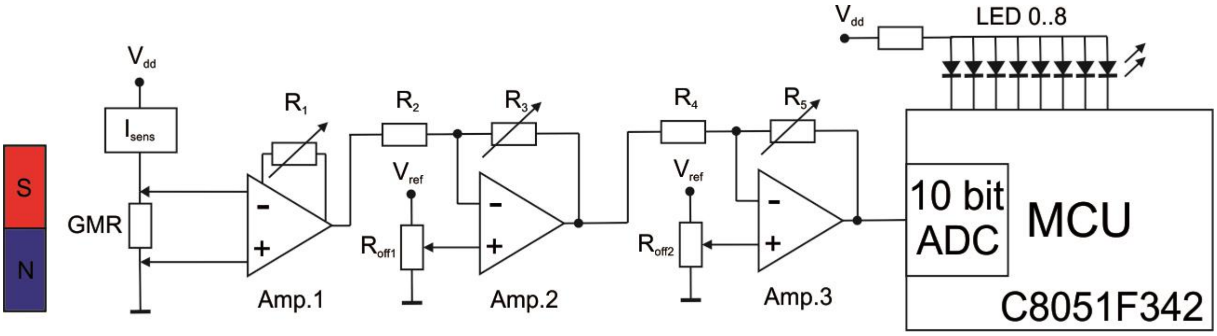

Supplementary Figure 5 | Signal conditioning and amplifying circuit. The figure displays the circuit diagram of the self-made acquisition device used in the demonstration of the on-skin magnetic proximity sensor shown in Fig. 1j,k,l and Supplementary Movie 4. Electrical connection of the sensor was performed in a two point's measurement. The sensor's response to an applied magnetic field was detected by measuring the changes in its resistance while approaching a neodymium magnet. The sensor was biased by a constant current of I_{sens} = 1 mA, supplied by the constant current generator circuit implemented directly on the acquisition device. The voltage signal from the sensor was amplified by an instrumentation amplifier (Amp. 1) with a gain defined by R_1. Subsequently, the signal passes a two cascade amplifier based on two operation amplifiers OPA354 (Amp. 2 & 3). Offset and gain of each cascade can be tuned (R_3, R_{off1}, R_5, R_{off2}) in order to fit the sensor signal to the input range of an analog to digital converter (ADC) embedded in a micro controller unit (MCU) C8051F342 of the acquisition device. The acquired data was processed in an MCU and displayed on a bar of 8 LEDs, to represent the detection of proximity between the magnetic sensor placed on the fingertip and the permanent magnet.

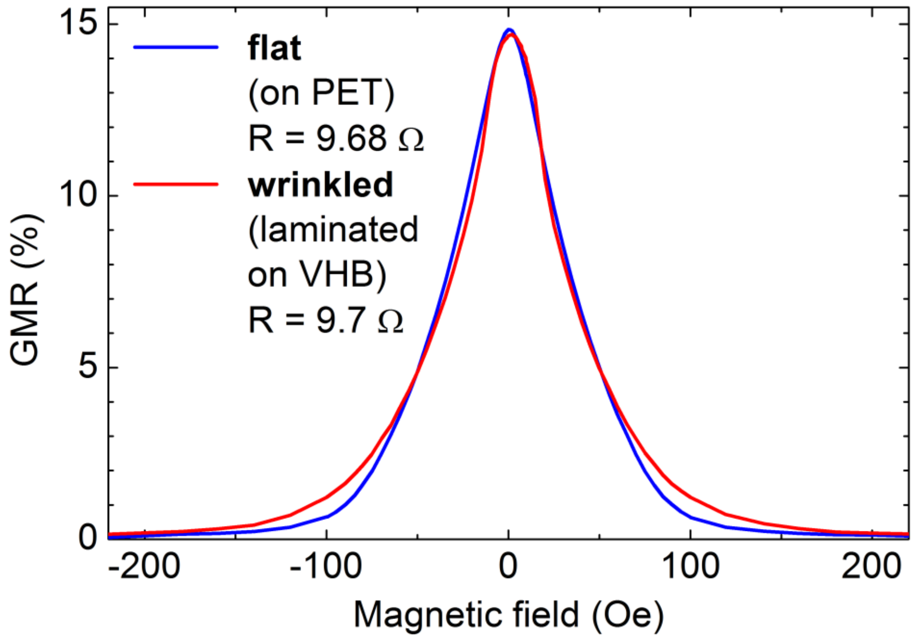

Supplementary Figure 6 | Influence of wrinkling on the GMR characteristic. The graph compares the GMR curves of a Py/Cu 2nd maximum multilayer at a flat state before lamination onto the pre-stretched stretchable support and at a wrinkled state after release of pre-strain (blue and red curve respectively). Since both curves show no significant difference, the influence of wrinkling on the GMR characteristic is small. Please note that this in only the case for uniaxial pre-strain and for wrinkles aligned parallel to the in-plane magnetic field applied for characterization.

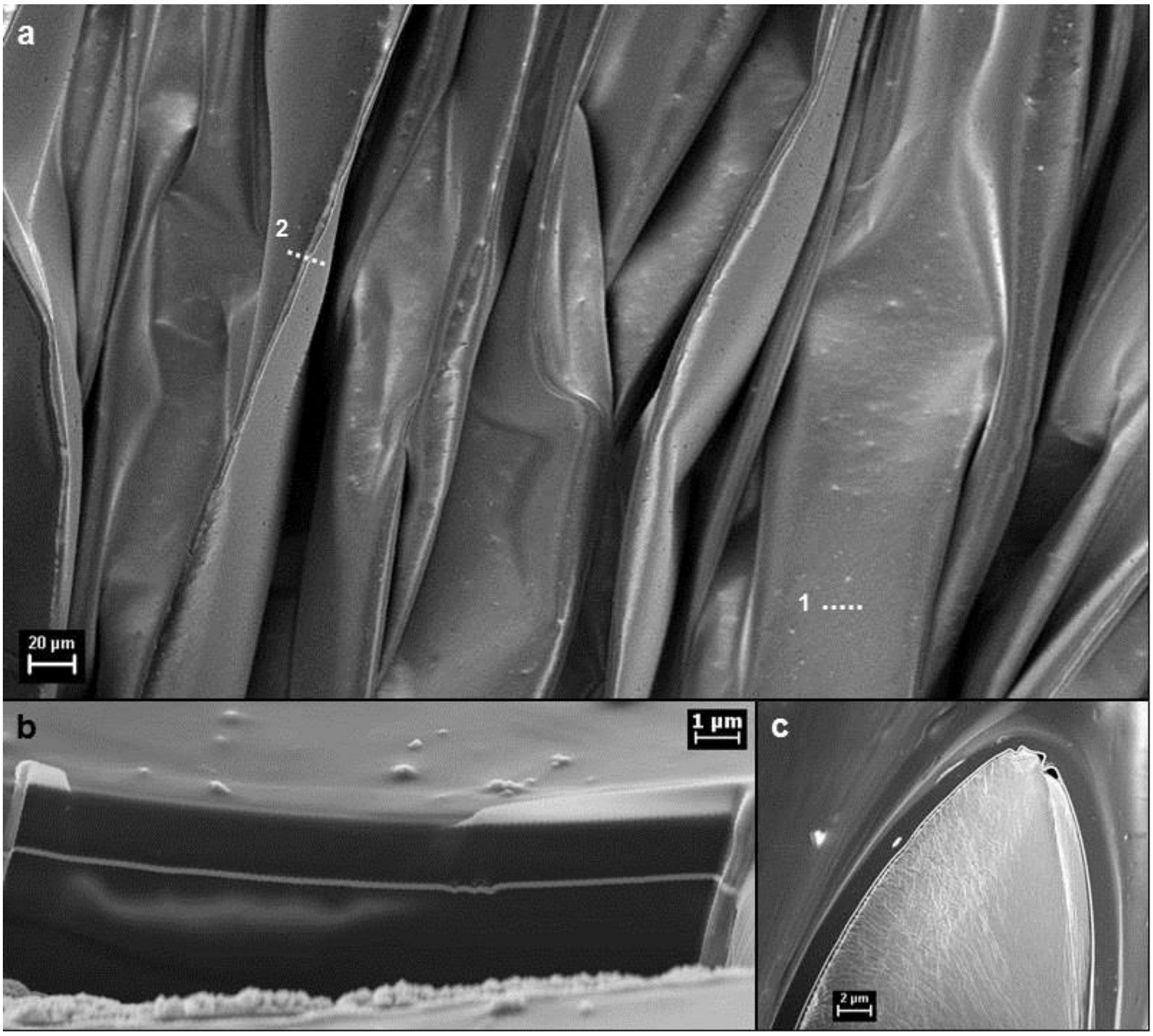

Supplementary Figure 7 | SEM investigation of wrinkled PET foils with GMR multilayers. The figure shows the stretchable GMR sensors after lamination onto a pre-stretched rubber tape and release of strain. (**a**) Top view on the highly wrinkled surface of the PET foil at a compression of 73% due to the release of pre-strain. The locations of the two FIB cuts through a flat (1) and highly bent (2) section of the sensor foil are indicated. (**b**) Cross-sectional view of the GMR layer (gray line) encapsulated between the VHB elastic tape (bottom) and PET foil (top). (**c**) Cross-sectional view of the tip of a wrinkle showing the high bending curvature of the magnetosensitive foil and its good adhesion to the stretchable support. Both pictures (b,c) correspond to Fig. 2d of the main manuscript.

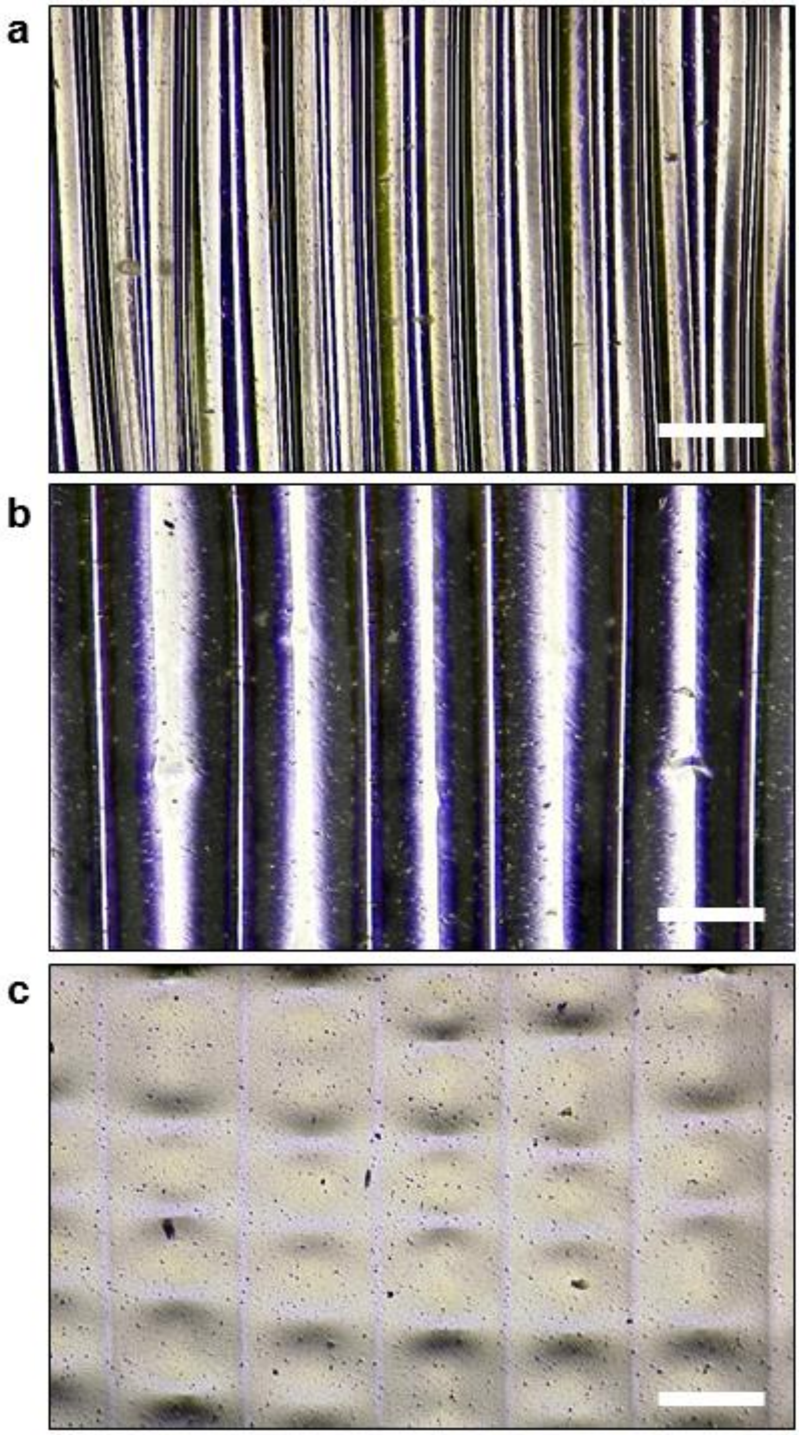

Supplementary Figure 8 | Optical micrographs of the stretchable GMR sensor at different strains. The images were obtained by a VK-X200 confocal microscope (Keyence) using the depth composition option of the white light observation mode. The pre-strain of the rubber support was 100% and the PET foil was laminated face up for increased reflectivity of the metalized layer on top. The GMR sensor surface is shown for low strain, high strain and for overstretching slightly above the maximum strain in (**a**), (**b**) and (**c**), respectively. The perpendicular buckles that occur in (**c**) are caused by transversal Poisson contraction, which laterally compresses the PET foil for tensile deformations above the strain it was laminated on the VHB tape. All scale bars correspond to 200 µm.

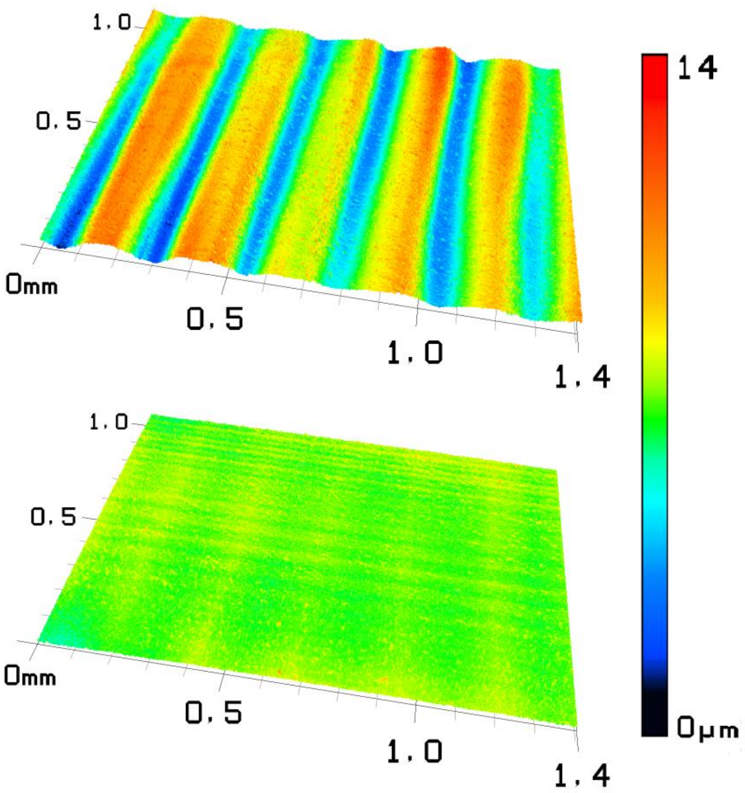

Supplementary Figure 9 | Confocal micrographs of the wrinkled and stretched GMR sensor surface. The images were recorded on a VK-X200 3D confocal laser scanning microscope (Keyence) and show the surface of the stretchable GMR sensors (metalized side up) at a stretched state just before reaching the maximum strain (top) and precisely at the maximum strain, above which the resistance starts to increase (bottom).

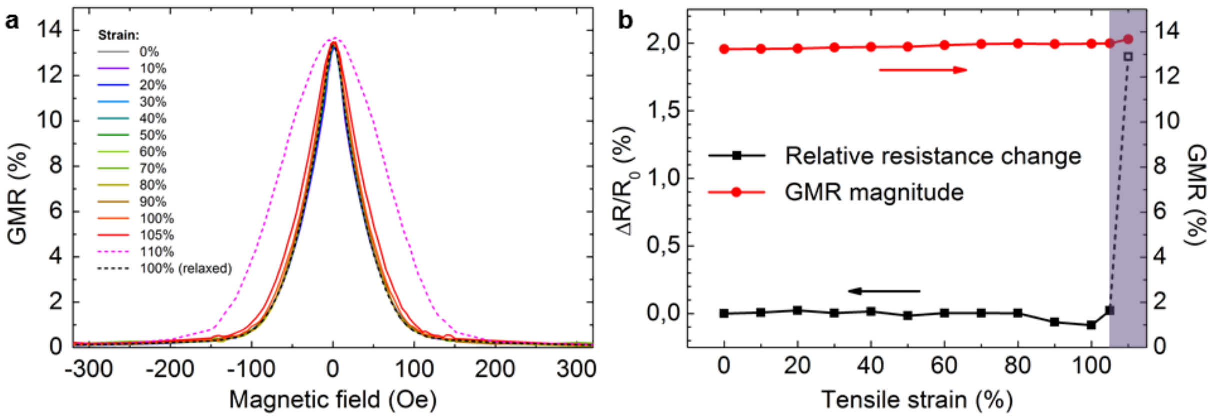

Supplementary Figure 10 | Overstretching of the GMR multilayer element. The data shows the results of a stretching experiment on a sample prepared with 150% pre-strain. (**a**) The GMR curves for different applied strains and (**b**) the GMR magnitude and relative resistance change over the applied strain. All data shows qualitatively the same behavior as in Fig. 3a,b. In (**a**), the GMR characteristic from the overstretched sample with increased resistance (at 110% strain, shaded area in (**b**)) is shown in addition, which gives an increased saturation field (dashed purple line). We attribute this transition to the occurrence of perpendicular buckles tensile deformations above the maximum strain, as shown in Supplementary Fig. 7c. The locally tilted plane at the sides of these buckles with respect to the applied magnetic field, causes increased switching fields at these locations, which shifts their magnetic saturation. This change in the GMR behavior is fully reversible, as the curve is in agreement with the previous ones, as the sensor is relaxed back to 100% strain (dashed black line).

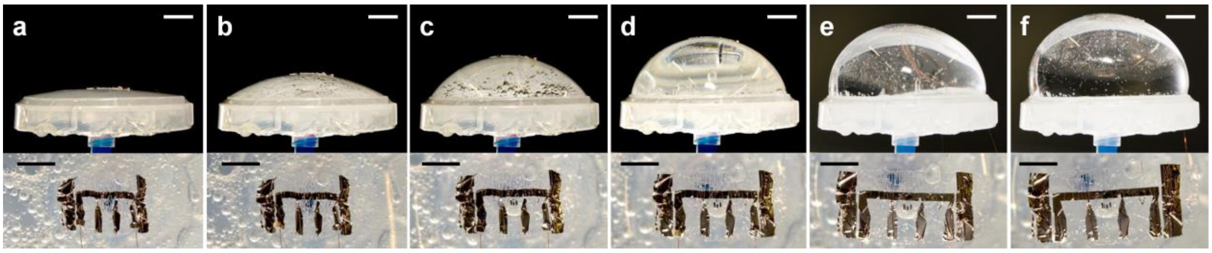

Supplementary Figure 11 | Inflating diaphragm and respective biaxial stretching of a GMR sensor. The top images show side-view photographs of the VHB diaphragm at different inflated states, according to the measurements presented in Fig. 4c,d. (all scale bars: 10 mm) These pictures were taken to estimate the areal strain at the top of the bulged diaphragm, where the GMR senor is mounted: The ratio of lengths of the meridian (along the photographic plane) on the bulged membrane surface l_m and the distance of its two fix edge points (i.e. the diameter of the plastic tray edge) l_d gives the total elongation of the meridian. Assuming rotational symmetry of tensile strains in the diaphragm surface around its axis, the respective areal strain at the top point is given by $(l_s/l_d)^2$-1 and result in: (**a**) 0%, (**b**) 11%, (**c**) 36%, (**d**) 78%, (**e**) 130% and (**f**) 175%. Bottom images show respective close-up photographs of the laterally expanding sensor on the diaphragm surface. (all scale bars: 5 mm)

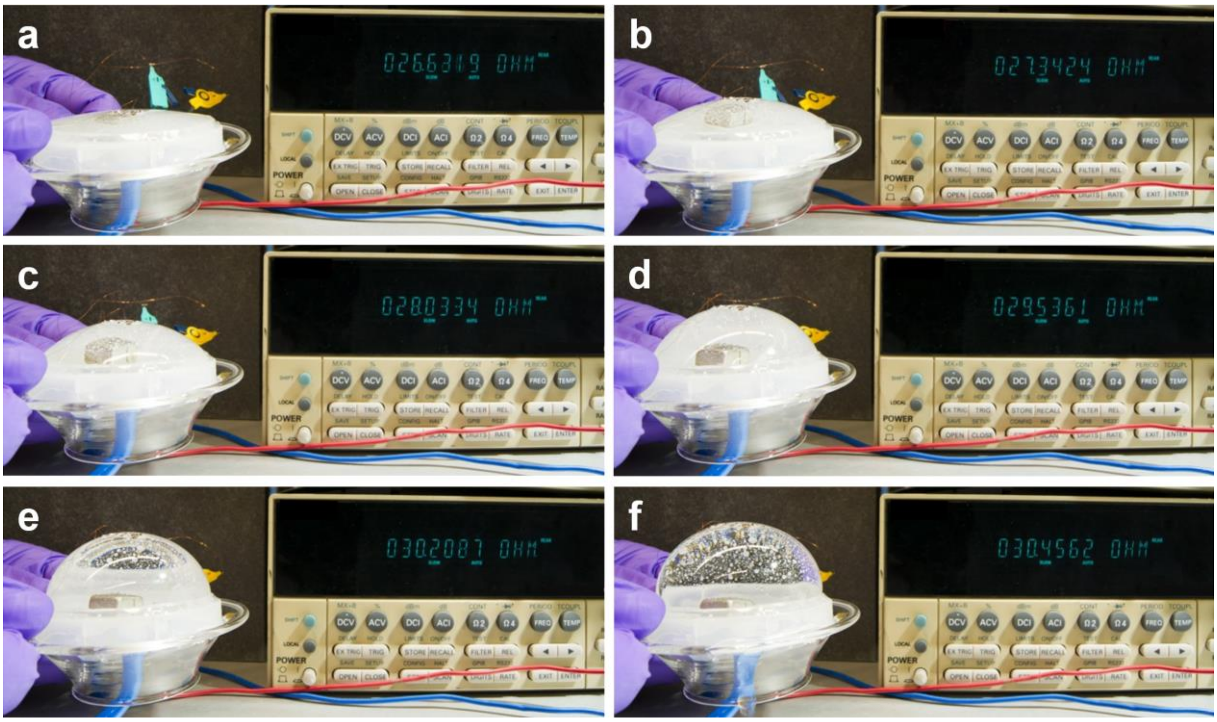

Supplementary Figure 12 | Magnetic detection of diaphragm inflation. Co/Cu 2nd maximum GMR sensor on a VHB diaphragm connected to a multimeter to display its resistance at different inflated states. The resistance change is induced by the increasing distance of the strain invariant sensor on the top point of the diaphragm to a permanent magnet fixed on the rigid frame below it.

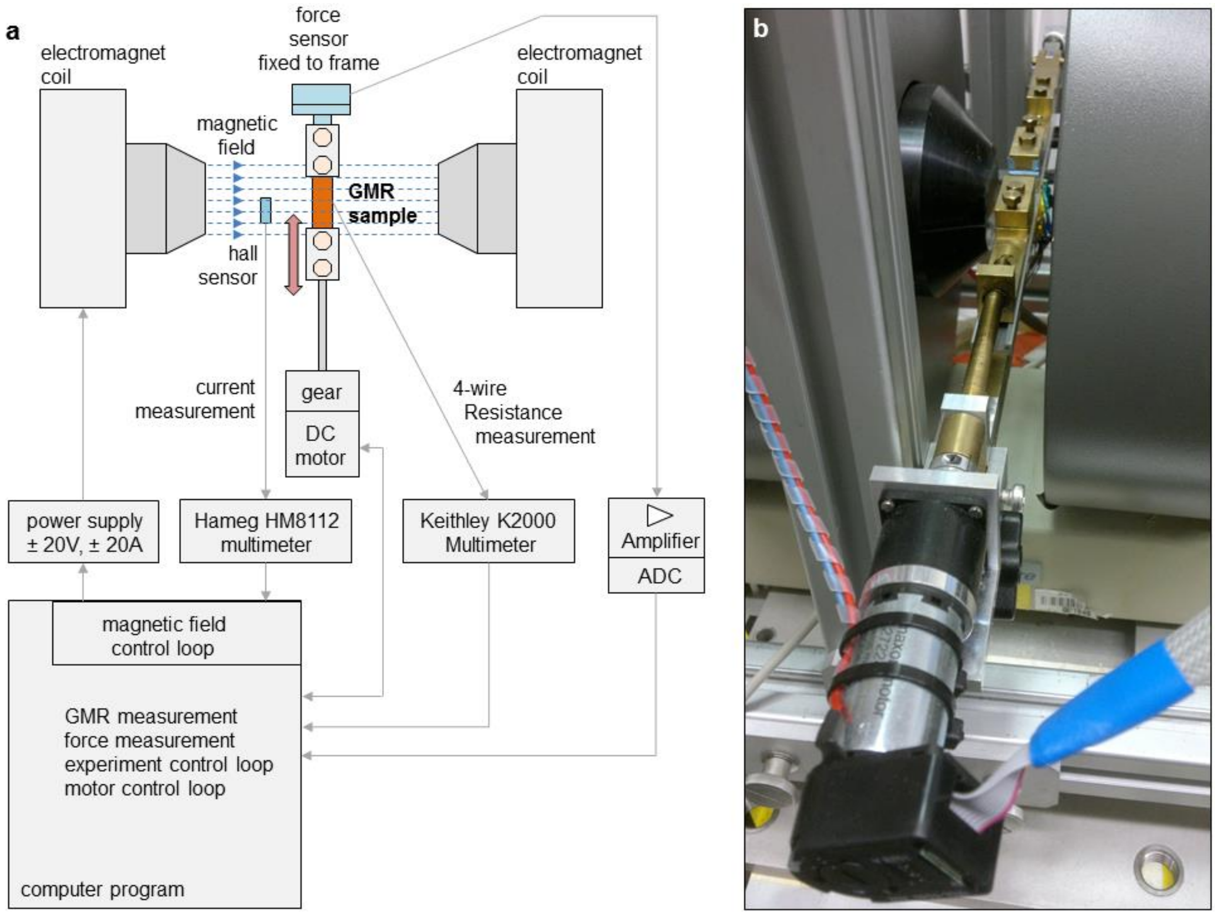

Supplementary figure 13 | The in-situ GMR stretching setup. (**a**) Schematic view of the setup. (**b**) Photograph of the motorized stretching stage between the pole shoes of the electromagnet. A close up of a contacted sample between the clamps can be found in Fig. 2b and Supplementary Movie 5.
